# Supplementary material for: Comparative multiomics analysis of cell physiological state after culture in a basket bioreactor
Source: Sci Rep. 2022 Nov 23;12:20161. doi: 10.1038/s41598-022-24687-4 (PMC9686226; doi:10.1038/s41598-022-24687-4)
Supplement: Supplementary file 1 — Supplementary Information 1. [file 41598_2022_24687_MOESM1_ESM.zip › raw data/Transcriptomic raw data/7.AS/2.dasplot/TE1vsTC1/A5SS/9_ATXN2L_NW_023666068.1_3341801_3341990_+@NW_023666068.1_3341801_3342044_+@NW_023666068.1_3342619_3343106_+.pdf]

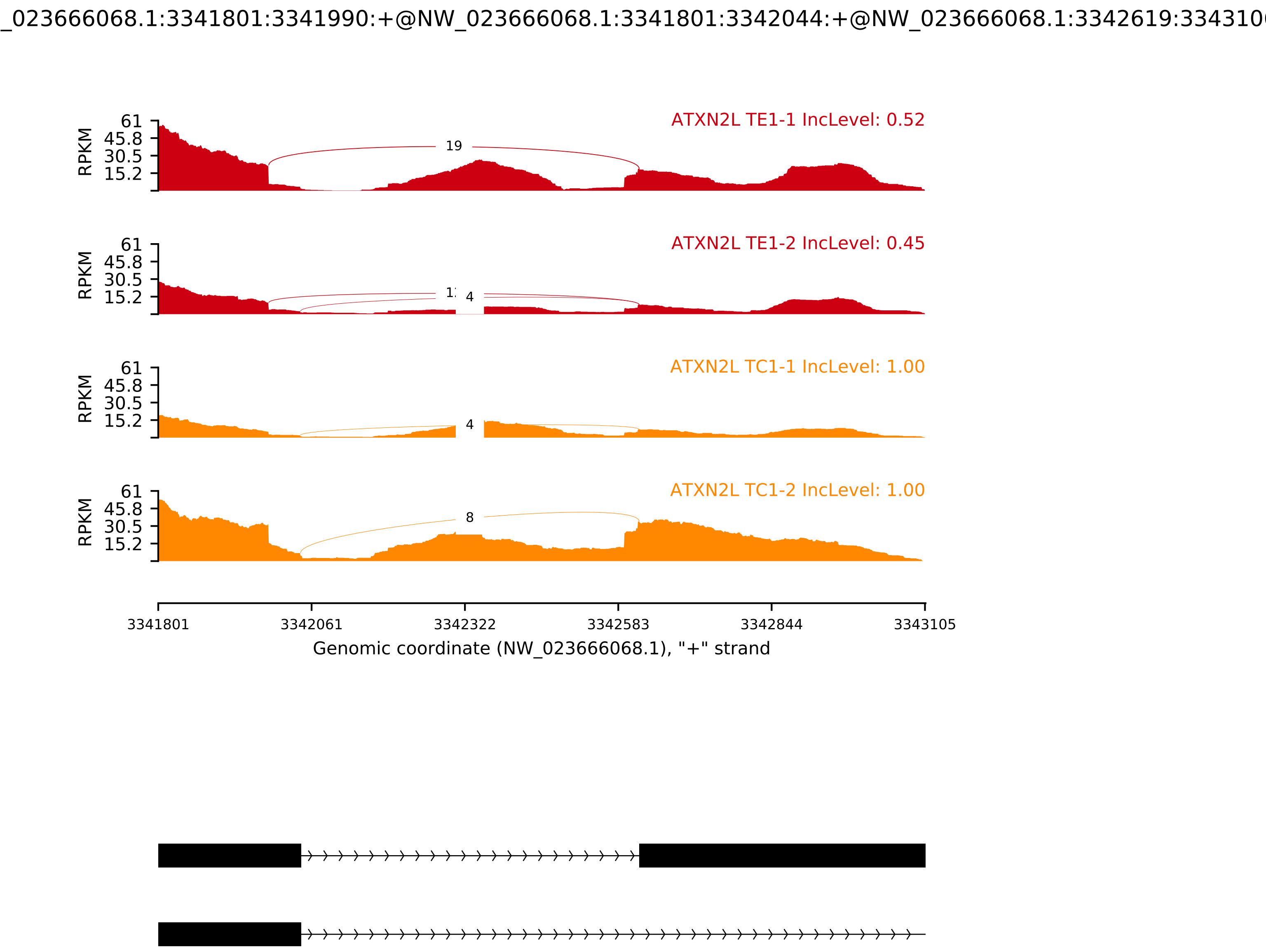

_023666068.1:3341801:3341990:+@NW_023666068.1:3341801:3342044:+@NW_023666068.1:3342619:334310
ATXN2L TE1-1 IncLevel: 0.52
19
ATXN2L TE1-2 IncLevel: 0.45
4
ATXN2L TC1-1 IncLevel: 1.00
4
ATXN2L TC1-2 IncLevel: 1.00
8
RPKM
61
45.8
30.5
15.2
3341801
3342061
3342322
3342583
3342844
3343105
Genomic coordinate (NW_023666068.1), "+" strand
